# Outcomes of endocardial versus endo-epicardial catheter ablation for ventricular arrhythmia in patients with arrhythmogenic right ventricular cardiomyopathy: a meta analysis

**DOI:** 10.1007/s10840-026-02348-0

**Published:** 2026-05-11

**Authors:** Ashish Yadav, Jacqueline Mirza, Eisha Gupta, Akshat Banga, Sawai Singh Rathore, Mohamed Ashraf Ahmed, Abhishek Maan, E. Kevin Heist

**Affiliations:** 1 University of Toledo, Toledo, Ohio USA; 2 Icahn School of Medicine at Mount Sinai, New York, NY USA; 3 Zucker Hofstra School of Medicine, Northwell Staten Island University Hospital, New York, NY USA; 4 Mount Auburn Hospital, Harvard Medical School, Cambridge, Massachusetts USA; 5 Ascension St Vincent Hospital, Milwaukee, WI USA; 6 Massachusetts General Hospital, Harvard Medical School, Boston, Massachusetts USA

**Keywords:** Endo-epicardial ablation, Endocardial ablation, Arrhythmogenic right ventricular cardiomyopathy, Ventricular arrhythmia, Major complications

## Abstract

**Background:**

Ventricular tachycardia (VT) ablation in arrhythmogenic right ventricular cardiomyopathy (ARVC) is typically performed using an endocardial first approach. However, epicardial involvement is common and recurrence remains frequent. We therefore compared the pooled efficacy and safety of endocardial-only versus combined endocardial and epicardial ablation strategies.

**Methods:**

We performed a systematic review and Bayesian meta-analysis of studies comparing endocardial and endo-epicardial VT ablation in patients with ARVC, including single- and double-arm designs. Ventricular arrhythmia (VA) recurrence was the primary endpoint and major complications were secondary. Bayesian methods were used to estimate pooled odds ratios (ORs) with 95% credible intervals (CrIs), with posterior predictive checks assessing model fit. A frequentist random-effects meta-analysis of double-arm studies was also conducted. Individual patient data (IPD) reconstructed from Kaplan–Meier curves enabled pooled survival analyses and hazard ratio (HR) estimation for VA recurrence.

**Results:**

Seventeen studies including 976 patients reporting primary or secondary outcomes were analyzed. Endo-epicardial ablation in ARVC was associated with a significantly lower risk of ventricular arrhythmia (VA) recurrence (Bayesian OR 0.47, 95% CrI 0.34–0.64), with similar frequentist analysis results (OR 0.48, 95% CI 0.30–0.75, *p* = 0.001). Reconstructed IPD from 460 patients showed reduced VA recurrence (HR 0.39, 95% CI 0.28–0.52; log-rank *p* < 0.001). Across 15 studies (866 patients), the combined approach increased complication risk (Bayesian OR 6.28, 95% CI 2–24). The frequentist model demonstrated a comparable increase (OR 3.19, 95% CI 1.02–10, *p* = 0.047).

**Conclusion:**

Endo-epicardial ablation reduced VA recurrence but increased major complication risk compared with endocardial ablation.

**Supplementary Information:**

The online version contains supplementary material available at https://doi.org/10.1007/s10840-026-02348-0.

## Background

The current understanding of the arrhythmogenic substrate, as well as the preferred ablation approach, for ventricular tachycardias (VTs) in patients with arrhythmogenic right ventricular cardiomyopathy (ARVC) has evolved rapidly. ARVC is an inherited disease, characterized by the fibrofatty replacement of native myocardium which creates a nidus for the formation of arrhythmogenic scar, and provides a substrate for occurrence of ventricular arrhythmias (VAs) through both triggered and re-entrant mechanisms [1]. Pharmacologic management of VAs in ARVC patients has demonstrated only limited efficacy in decreasing arrhythmia burden and does not directly affect the underlying substrate [2]. Catheter ablation has more recently emerged as a means of providing longer-term VA control, with the earliest approaches targeting the endocardial substrate [3, 4]. However, the endocardial-only approach often yields suboptimal mitigation of ventricular arrhythmias, which further necessitates the need for a subsequent epicardial approach to achieve better outcomes. With improvements in safety and approach of epicardial access, arguments have been made for a primary endo-epicardial approach for ablation of VAs in patients with ARVC [5, 6]. Although this has the potential to provide a durable VA ablation outcome in comparison with an endocardial-only approach, a combined approach is also associated with an increased risk of complications, including pericardial effusion, cardiac tamponade and damage to adjacent structures such as the phrenic nerve and coronary arteries [7, 8].

Although there are data from single-centre studies on both these approaches, there is a paucity of data on direct comparison of these two approaches for ablation of VAs in patients with ARVC [6, 9]. This meta-analysis aims to compare endocardial-alone and endo-epicardial approach to VA ablation in ARVC patients.

## Methods

This systematic review and meta-analysis was designed, conducted, and reported according to the Preferred Reporting Items for Systematic Review and Meta-Analysis (PRISMA) 2020 guidelines [10] (Supplementary Table S1).

### Search strategy and study selection

A systematic literature search of major bibliographic databases (PubMed, Embase, Google Scholar, Scopus and Cochrane Library) was conducted from database inception until August 2025. The search strategy used keywords such as:

- Endo-epicardial Ablation
- Endocardial Ablation
- Ventricular Arrhythmia
- Arrhythmogenic Right Ventricular Cardiomyopathy

These keywords were used in combination with the Boolean operators such as “AND” and “OR” (Supplementary Table S2). We did not apply any filters based on the language and any year of publication. The search was aimed at identifying studies that had evaluated recurrence of VA in patients with ARVC who underwent either endo-epicardial ablation or endocardial ablation. 

#### Inclusion criteria (PICOS format)

(i) P (Population): Arrhythmogenic right ventricular cardiomyopathy
(ii) I (Intervention): Endo-epicardial catheter ablation
(iii) C (Comparator): Endocardial only catheter ablation (for double-arm studies)
(iv) O (Outcomes): Ventricular arrhythmia/recurrence and major complications
(v) S (Study Design): Randomized controlled trials or observational studies, including both double-arm and single-arm studies

#### Exclusion criteria

We excluded, (a): studies where catheter ablation was combined with another major intervention; (b): if the study design was that of case report, case series, review article, conference abstract, editorial or viewpoint; (c): studies that did not report any of the predefined outcomes of interest, such as the VA recurrence. After the removal of duplicates, two investigators (A.Y. and A.B.) screened all articles using titles and abstracts and excluded those that did not adhere to our predefined inclusion criteria. Subsequently, the full texts of the shortlisted articles were comprehensively assessed for inclusion in the meta-analysis. Any discrepancies or disagreements were resolved by discussion with a third investigator (S.S.R.) to reach a consensus. All the reference lists were manually screened to identify additional studies.

### Data extraction and quality assessment

Two investigators (A.Y. and A.B.) extracted the following data: first author’s name, study design, mean age, total number of patients, patient demographics and study period. A third investigator (S.S.R.) independently crosschecked the data extraction to ensure accuracy and validity. For the purpose of quality assessment, two investigators (A.Y. and A.B.) independently utilized the Risk of Bias in Non-Randomized Studies of Interventions (ROBINS-I) [11]. ROBINS-I is a standard method to assess the risk of bias across 7 domains, including confounding, selection of participants, classification of interventions, deviations from intended interventions, missing data, measurement of outcomes, and selection of the reported result.

### Study overview

We performed a pooled analysis to evaluate outcomes for endocardial only versus the combined approach of endo-epicardial ablation in ARVC patients. The Bayesian arm-based meta-analysis incorporating data from both double-arm and single-arm studies was performed to evaluate the odds of VA recurrence and major complications. To provide a complementary sensitivity analysis, we also performed frequentist, random effects meta-analysis which only incorporated double-arm studies. For time-to-event evaluation, only double-armed studies reporting Kaplan-Meier curves were used to reconstruct individual patient data (IPD) and analyze recurrence free survival. All analyses were conducted using R version 2024.12.0.467.

### Data synthesis and analysis

To incorporate evidence from all the available studies, the Bayesian meta-analysis including both single-arm and double-arm studies was performed. We applied an arm-based binomial model with a logit link and random effects to account for between-study variability. Posterior estimates were summarized as medians with 95% credible intervals and the overall odds ratio comparing endo-epicardial with endocardial ablation was derived from these estimates. Model convergence was assessed using standard diagnostics and model fit was evaluated with posterior predictive checks. Results were presented as pooled estimates and visualized using a summary plot of the posterior odds ratio and its uncertainty.

We carried out a traditional frequentist meta-analysis limited to double-arm studies as well. For this, we analyzed raw event counts using the *meta* package in R and its *metabin ()* function, applying a random-effects model based on the Der Simonian–Laird method with the log odds ratio as the effect measure. A continuity correction was applied when studies reported zero events. Results were presented in RevMan-style forest plots, showing odds ratios with 95% confidence intervals for individual studies, the pooled estimate, study weights, and measures of heterogeneity (I², τ² and p-value), along with the overall effect p-value. To further evaluate robustness and to evaluate whether one study influenced the overall pooled effect estimates, we also performed a “leave-one-out” sensitivity analysis by sequentially omitting each study.

For the survival analysis, we only used double-arm studies that had Kaplan–Meier curves available in the published manuscript. These plots were digitized with WebPlotDigitizer (v4.5), to provide survival probabilities for both treatment groups over time. If a study had the data available on the numbers-at-risk, those were considered to make the reconstruction more reliable. With these inputs, we rebuilt pseudo–individual patient data (IPD) using the approach described by Liu et al. implemented in the *IPDfromKM* package in R [12]. To check how well the reconstructed curves matched the originals, we compared them using several error measures, including mean absolute error (MAE), root mean square error (RMSE) and maximum absolute error (MaxAE). Reconstructions were considered acceptable, if the RMSE and MAE were no higher than 0.05. We also applied Mann–Whitney and bootstrap t-tests to assess whether the reconstructed and original survival distributions were statistically different, where a large p value is desirable.

We combined the reconstructed IPD from all eligible double-arm Kaplan–Meier studies into a single dataset. Kaplan–Meier curves were then used to estimate recurrence-free survival for VA, and the two treatment groups were compared with the log-rank test. Hazard ratios (HRs) with 95% confidence intervals (CIs) were calculated using Cox proportional hazards regression, with both pooled analyses and models stratified by study. The proportional hazards assumption was examined using Schoenfeld residuals. Median survival times with 95% CIs were reported and when the survival curve did not reach the 50% threshold, the result was noted as “not reached” (NR). Survival plots were drawn with shaded confidence bands and log-rank p-values displayed. Cumulative hazard curves were also produced from the pooled data. The pooled cumulative hazard curves were presented for descriptive visualization of risk accumulation over time. To further examine temporal trends in ventricular arrhythmia recurrence free survival, graphical visualization was performed using GraphPad Prism (GraphPad Software, San Diego, CA, USA). To evaluate the overall efficacy and the risk of complications, we also assessed number needed to treat (NNT) and number needed to harm (NNH) for both the ablation strategies. The NNT or NNH was calculated as the reciprocal of the absolute risk difference between groups, derived from pooled event rates, with directionality determined by whether the intervention reduced or increased the event risk.

## Results

### Systematic literature search results

**Fig. 1:**
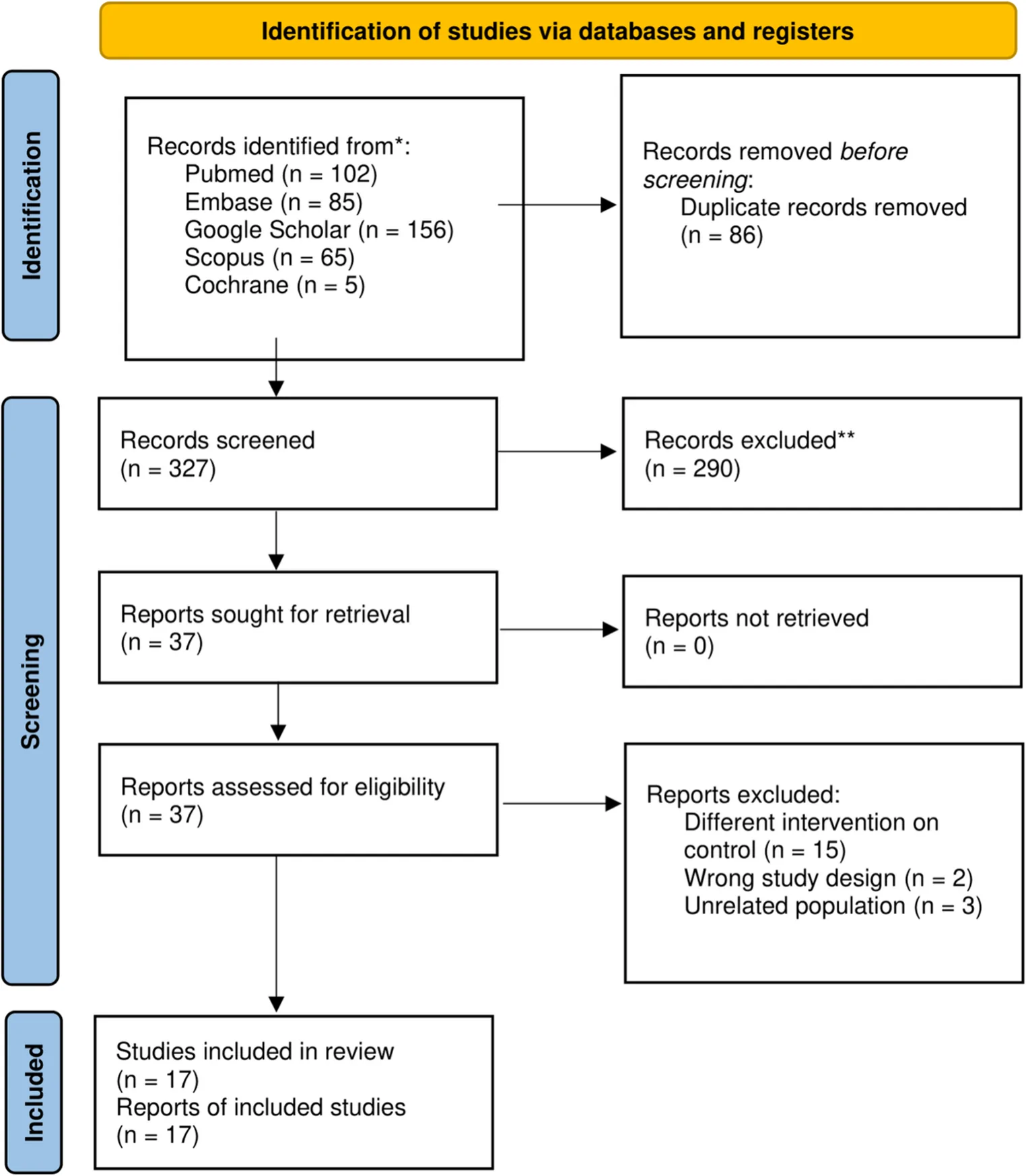
The Preferred Reporting Items for Systematic Reviews and Meta-Analyses (PRISMA) 2020 flowchart

Our preliminary search identified a total of 413 articles. After removing the duplicates (*n* = 86), the remaining 327 articles were screened for title and abstract. After excluding the articles that did not meet the inclusion criteria (*n* = 290), the remaining 37 articles were subjected to a comprehensive full-text assessment, including a manual search of the reference lists of all studies. Twenty studies were excluded for the following reasons: different intervention or control (*n* = 15), did not report primary outcome (*n* = 2) and unrelated population (*n* = 3). For our final analysis, 17 studies were included. The systematic literature search process using the PRISMA flowchart is shown in (Fig. 1).

### Baseline study characteristics

This meta-analysis included 17 studies published between 2005 and 2024. The study enrolled a total of 976 participants. Among the included studies, 6 studies were single-armed studies while 11 were double-armed studies. The study designs included both prospective and retrospective observational studies **(**Table 1**).**

**Table 1:** Baseline demographics of patients in studies included in meta-analysis

Study	Mean Age	*n*	Male (*n*)	Ablation approach		Number of VTs		HF		Mean LVEF (%)	Number of patients with ICD	Mean follow up time (months)	AAD at follow up status	Ablation extent	Year	Epicardial access strategy	KM available?	Mapping
				Endo	Endo-epi	Endo	Endo-epi	Endo	Endo-epi									
Marchlinski et al. [4]	47 ± 18	21	18	21	NR	NR	NR	5	NR	55	21	27	Predominantly ON AAD	Extensive substrate modification	2004	NR	No	VT mapping
Verma et al.[3]	41 ± 15	22	15	22	NR	3 ± 2	NR	1	NR	55	22	35.8	Predominantly ON AAD	Extensive substrate modification	2005	NR	Yes	Substrate mapping
Dalal et al. [13]	36 ± 9	24	11	24	NR	NR	NR	0	NR	Normal	19	32	Predominantly ON AAD	Focal /VT-guided ablation	2007	NR	Yes	Conventional VT mapping
Bai et al.[6]	35.4 ± 12.6	49	33	23	26	NR	NR	NR	NR	55	49	36.5	Predominantly OFF AAD	Extensive substrate modification	2011	Hybrid staged	Yes	VT + substrate mapping
Philips et al.[14]	38 ± 13	87	45	64	23	NR	NR	NR	NR	NR	83	88.3	No protocol	Focal / VT-guided	2012	Failure staged	Yes	Substrate mapping
Berruezo et al. 2012 [15]	42 ± 13	11	9	NR	11	8.9 ± 7.2	16.5 ± 5.4	NR	NR	NR	6	13.7	Predominantly ON AAD	Extensive substrate modification	2011	Upfront	No	VT + substrate mapping
Santangeli et al. 2015 [16]	39 ± 15	62	45	23	39	NR	NR	NR	NR	NR	58	56	No protocol	Extensive substrate modification	2015	Failure staged	Yes	VT + substrate mapping
Berte et al. [17]	55 ± 16	20	16	14	6	NR	NR	NR	NR	51.8	7	17.8	No protocol	Limited substrate-based	2016	Recurrence staged	No	VT + substrate mapping
Müssigbrodt et al. [18]	53.4 ± 14.2	45	30	23	22	1.9 ± 1.4	2.5 ± 1.4	3	3	56	NR	31.3	No protocol	Focal / VT-guided	2017	Failure staged	Yes	VT + substrate mapping
Wei et al. [19]	39.9 ± 12.9	48	33	30	18	NR	NR	5		51.4	11	71.4	Predominantly ON AAD	Limited substrate-based	2017	Hybrid staged	Yes	VT + substrate mapping
Berruezo et al. 2017 [20]	42.3 ± 12.5	41	36	NR	41	NR	NR	NR	NR	51.3	31	32.2	Predominantly ON AAD	Extensive substrate modification	2016	Upfront	Yes	VT + substrate mapping
Santangeli et al. 2019 [21]	45 ± 13	32	23	9	23	NR	NR	NR	NR	61	0	46	No protocol	Extensive substrate modification	2019	Upfront	Yes	VT + substrate mapping
Mahida et al. [22]	38 ± 17	110 (75 ablation)	91	35	40	NR	NR	NR	NR	55	109	36	Predominantly ON AAD	Limited substrate-based	2019	Recurrence staged	Yes	VT + substrate mapping
Mathew et al. [23]	44 ± 16	47	38	26	21	NR	NR	NR	NR	NR	35	50	No protocol	Focal / VT-guided	2019	Failure staged	Yes	Substrate mapping
Gandjbakhch et al. [24]	44 ± 13	65	51	46	19	NR	NR	NR	NR	61	12	69.6	No protocol	Limited substrate-based	2021	Hybrid staged	Yes	VT + substrate mapping
Daimee et al. [25]	34.7	166	58	60	106	NR	NR	NR	NR	53.7	115	62.4	Predominantly OFF AAD	Limited substrate-based	2021	Selective staged	Yes	VT + substrate mapping
Chaumont et al. [26]	43.8 ± 16.8	74	56	74	NR	≥ 3	NR	17	NR	54.6	73	84.8	Mixed / Non-protocolized	Extensive substrate modification	2024	NR	Yes	VT + substrate mapping

Legend: Predominantly OFF AAD: Antiarrhythmic drugs were intentionally discontinued following ablation in the majority of patients

Predominantly ON AAD: Antiarrhythmic drugs were routinely continued following ablation

Focal / VT-guided ablation: Ablation was limited to clinically relevant ventricular tachycardia circuits

Limited substrate-based: Ablation combined VT-guided lesion delivery with targeted substrate modification, without complete scar homogenization

Extensive substrate modification: Ablation involved broad modification of arrhythmogenic substrate and in some cases near-complete scar homogenization

Upfront: Planned first-line combined endocardial and epicardial ablation

Failure staged: Epicardial access performed after unsuccessful endocardial ablation or persistent inducibility during the same procedure

Recurrence staged: Epicardial ablation performed in a separate procedure after VT recurrence following prior endocardial ablation

Selective staged: Epicardial access guided by arrhythmia characteristics (e.g., ECG morphology), not strictly dependent on endocardial failure

Hybrid staged: Combination of multiple approaches or unclear protocol with both failure-driven and staged/repeat-procedure pathways

Abbreviations: *LVEF* Left ventricular ejection fraction, *ICD* Implantable cardioverter defibrillator, *AAD* Anti arrhythmic drug, *VT* Ventricular tachycardia

### Outcomes

Our meta-analysis reports two major outcomes.

#### Ventricular arrhythmia recurrence

**Fig. 2:**
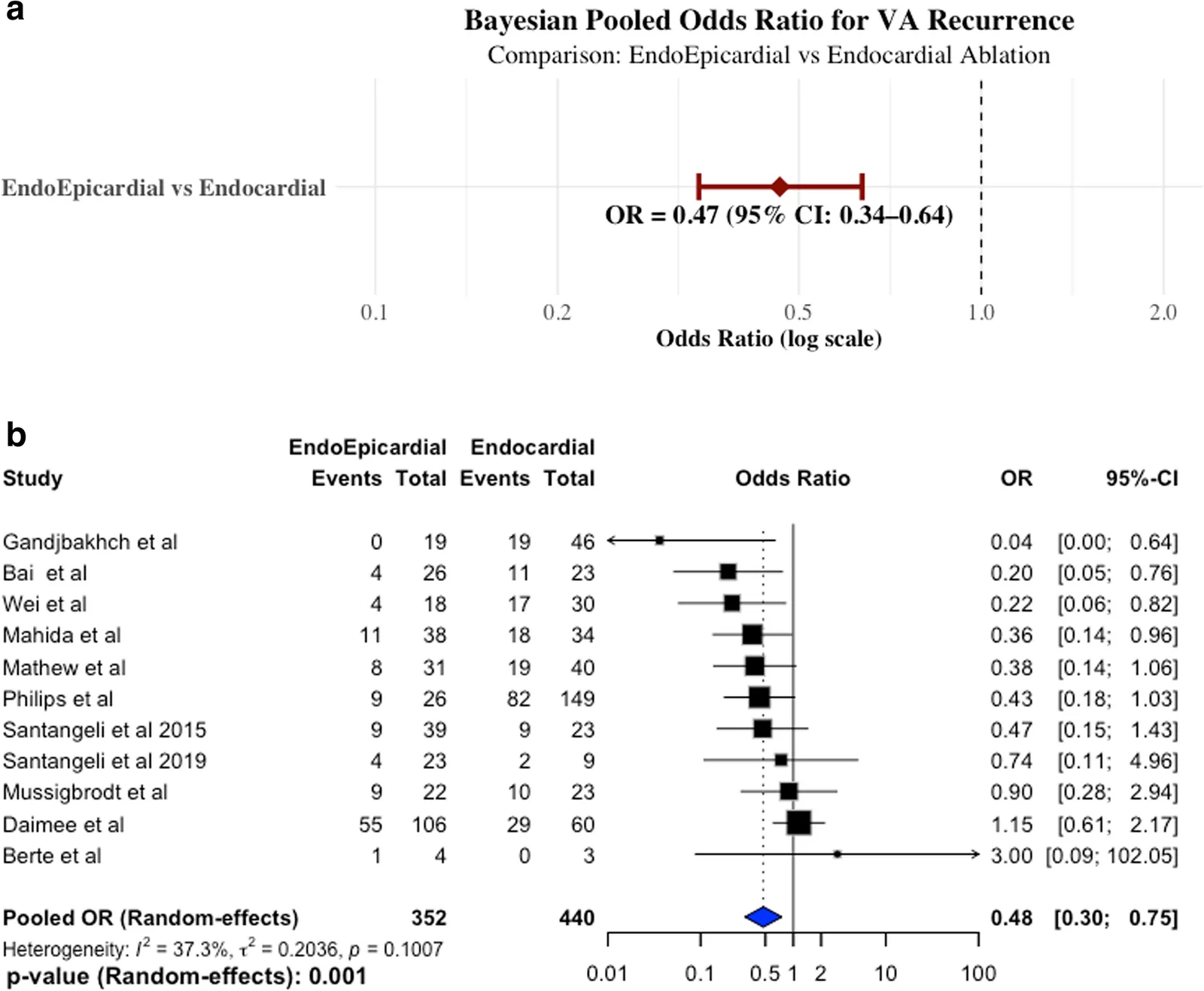
Panel** a**: Bayesian Forest plot of comparison of recurrence of ventricular arrhythmias between the Endocardial only and the Endo-Epicardial approach in ARVC. Panel **b**: Frequentist Forest plot of ventricular arrhythmia recurrence (double-arm studies only) between the Endocardial only and the Endo-Epicardial approach in ARVC.

Across 17 studies including 976 patients (397 endo-epicardial, 579 endocardial-only), VA recurrence was significantly reduced in the combined ablation group. In the Bayesian analysis, endo-epicardial ablation was associated with decreased odds of VA recurrence compared with endocardial-only ablation (Pooled OR = 0.47, 95% CrI: 0.34–0.64) (Fig. 2, Panel a). Based on the posterior predictive checks, we also observed adequacy of model calibration with the observed summary estimated that were within the central distribution of replicated datasets (Supplementary Fig. 1). Posterior estimates for recurrence probabilities in each treatment arm and the overall odds ratio are summarized in Supplementary Table 3.

**Fig. 3:**
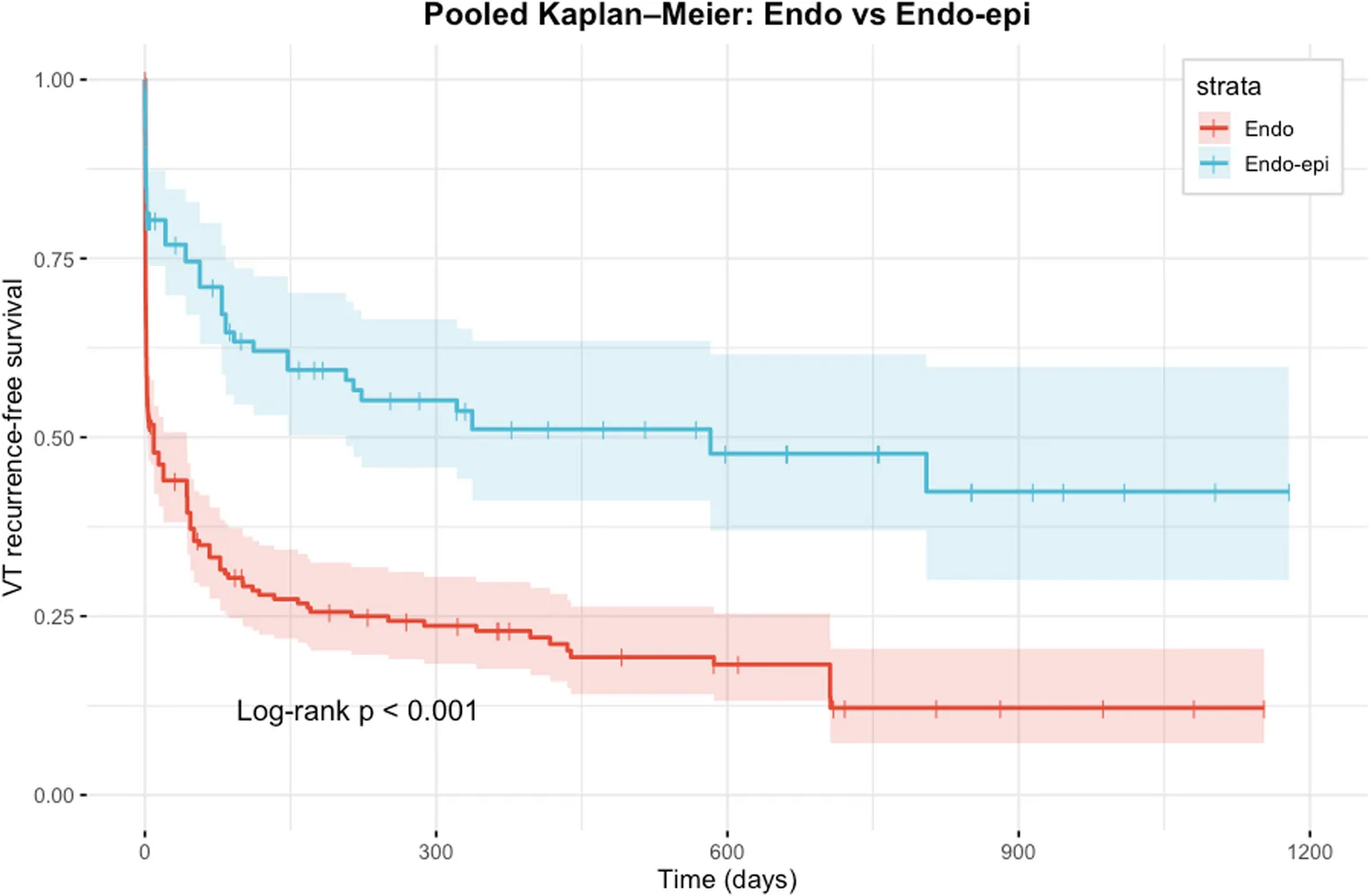
Pooled Kaplan–Meier curves for VA recurrence-free survival between the endocardial and the endo-epicardial ablation approaches

**Fig. 4:**
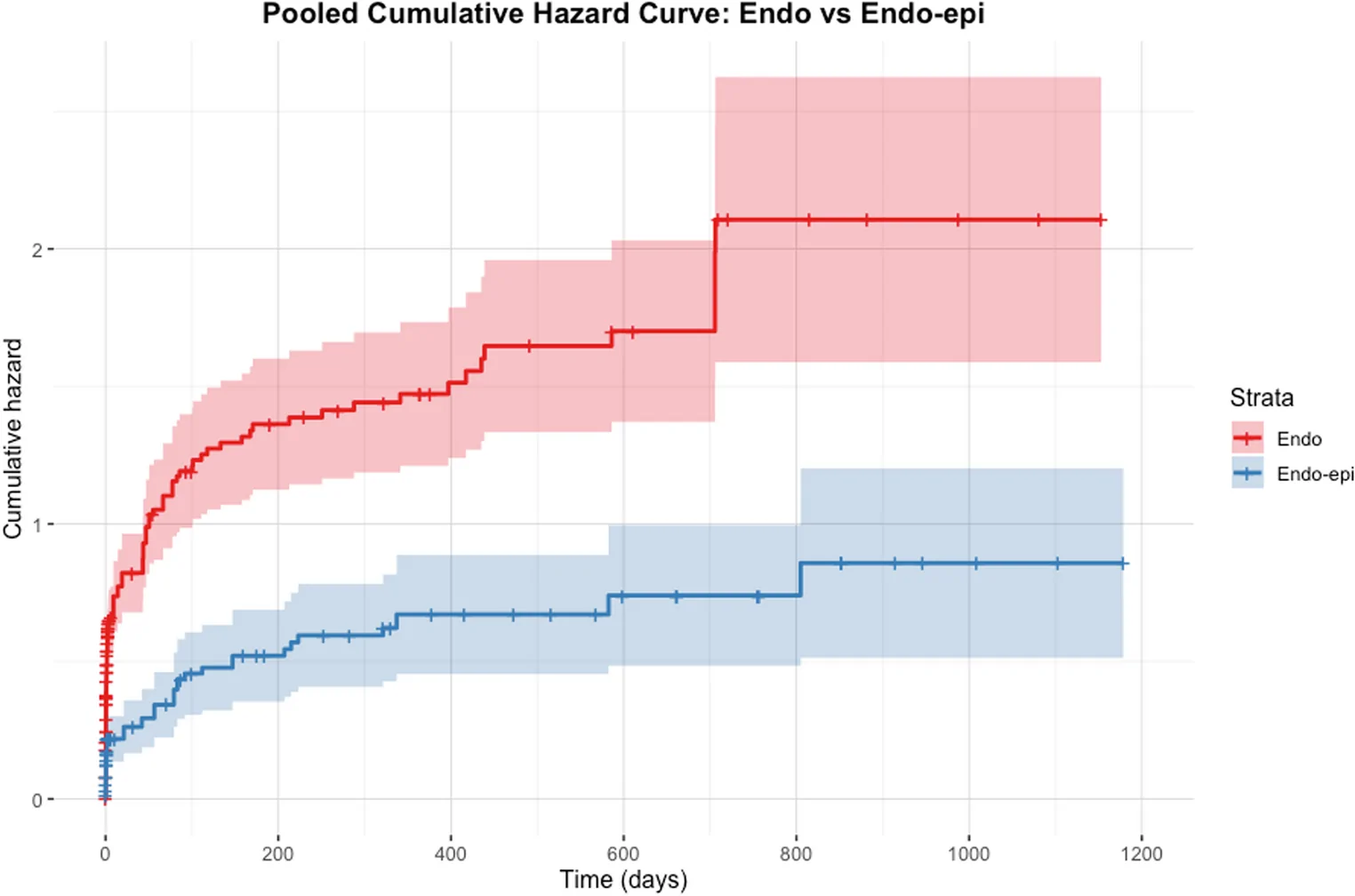
Pooled cumulative hazard curves for VA recurrence between the endocardial and the endo-epicardial ablation approaches

Similar to these findings, the frequentist random-effects model (which had only consisted of double-arm studies that had compared the 2 ablation strategies directly) also yielded superior efficacy of the combined approach in comparison to an endocardial-only approach for suppression of VAs (pooled OR of 0.48, 95% CI of 0.30–0.75, *p* = 0.001). The analysis with frequentist random-effects model had moderate degree of heterogeneity (I² = 37.3%) (Fig. 2, Panel b). Upon the “leave-one-out” sensitivity analysis, we observed that no single study in our analysis affected the overall estimate of risk-ratios (Supplementary Fig. 2). To further characterize VA recurrence-free survival, we also performed a pooled Kaplan–Meier analysis as well. This included data on a total of 460 patients (299 in the endocardial-only arm and 161 in the endo-epicardial arm). In this analysis, we also observed that the combined endo-epicardial ablation strategy was associated with superior outcomes regarding VA recurrence-free survival, as compared with the endocardial-only group (log-rank *p* < 0.001) (Fig. 3). In Cox regression, endo-epicardial ablation was associated with a 61% relative reduction in risk of VA recurrence (HR = 0.39, 95% CI: 0.28–0.52). Median VA recurrence-free survival was 489 days overall (95% CI: 378–587 days) in the endo-epicardial strategy and 338 days in the endocardial-only group (95% CI: 222–451 days), and this did not reach statistical significance (Supplementary Table 4). Pooled cumulative hazard curves demonstrated consistently lower cumulative hazard for the endo-epicardial group compared with the endocardial-only group (Fig. 4).

#### Major complications

**Fig. 5:**
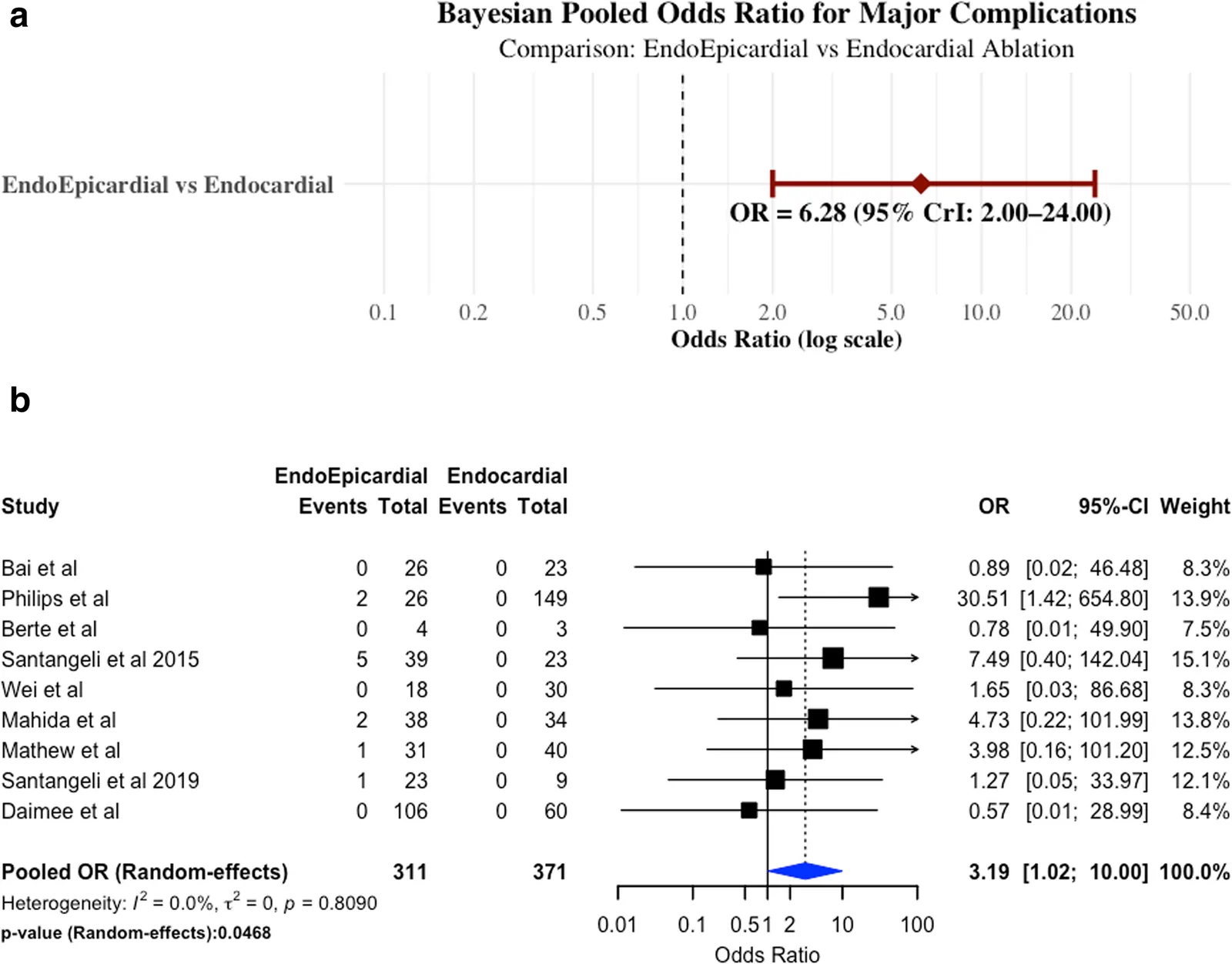
Panel** a**: Bayesian Forest plot of major complications between the Endocardial and the Endo-Epicardial ablation approaches. Panel** b**: Frequentist Forest plot of major complications (double-arm studies only) between the Endocardial and the Endo-Epicardial ablation approaches

Across 15 studies including 866 patients (356 in the endo-epicardial group vs. 510 in the endocardial-only group), complication rates were consistently higher in the combined endocardial-epicardial ablation group. Major complications were defined as events that were procedural or early post-procedural. The analysis focused on complications related to the ablation procedure or epicardial access. Late follow-up events not directly attributable to the procedure were excluded whenever distinguishable. The absolute complication rates were 0.4% (2/565) in the endocardial group compared with 3.9% (14/363) in the endo-epicardial group, supporting a higher procedural risk with the combined approach. In the Bayesian framework, endo-epicardial ablation was associated with an increased odds of major complications (OR = 6.28, 95% CrI: 2.00–24.00) (Fig. 5, Panel a). Posterior predictive checks demonstrated that the model adequately captured the observed distribution of complications (Supplementary Fig. 3). Posterior estimates for recurrence probabilities in each treatment arm and the overall odds ratio are summarized in Supplementary Table 5. The frequentist random-effects model, consisting only of double-armed studies, yielded a pooled odds ratio of 3.19 (95% CI: 1.02–10.0, *p* = 0.046), with no evidence of heterogeneity (I² = 0%) (Fig. 5, Panel b). Upon the leave-one-out sensitivity analysis, there was no single study that might have significantly altered the overall risk-ratio estimate (Supplementary Fig. 4).

### Quality assessment, publication bias and temporal trends

**Fig. 6:**
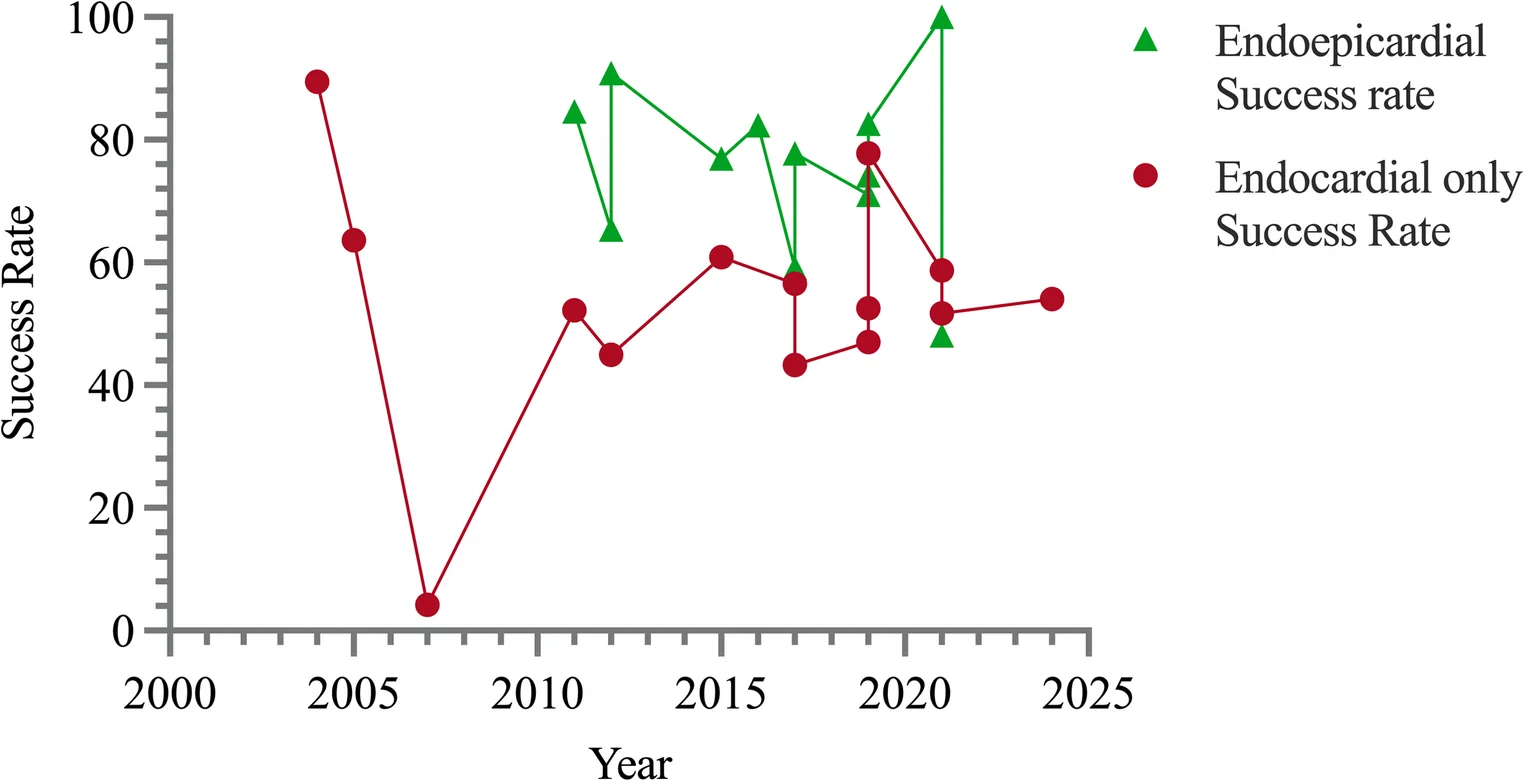
Temporal trends in success rates, defined as freedom from ventricular arrhythmia recurrence, comparing Endocardial only and Endo-Epicardial ablation approach

Based on our assessment using the ROBINS-I tool, majority of the studies that we had included in our analysis were concluded to have a relatively low risk of bias (Supplementary Figs. 5 and 6). The funnel plots for both the outcomes appeared to be visually symmetrical, which suggested a minimal risk of publication bias (Supplementary Figs. 7 and 8). We also analysed the temporal trends of success rates. Upon analysing these data on success rates, we did not observe a consistent improvement in the success rates of either approach for VT ablation in ARVC. These findings are summarized in Fig. 6.

## Discussion

Our meta-analysis observed the following key findings:

- A combined endo-epicardial approach for VT ablation in patients with ARVC was associated with a significant reduction in recurrence of VAs as compared to an endocardial approach alone.
- The combined approach was associated with a greater risk of major complications that included both peri-and post-procedural when compared with endocardial ablation alone.
- The NNT to prevent a single event of VT recurrence after endo-epicardial ablation was 6 and the NNH leading to a peri-procedural and post-procedural complication with this approach was 28.

Our comprehensive search was conducted using rigorous inclusion criteria, utilizing both PRISMA and ROBINS-I, and was analysed using both Bayesian and frequentist methods to enhance robustness. Our results were also confirmed by sensitivity analyses and had an overall low risk of publication bias. Taken together, these findings suggest that endo-epicardial ablation represents a more durable option as part of a stepwise approach which can improve VT ablation outcomes in patients with ARVC. This combined approach could be particularly useful in patients who continue to experience VT despite having had previous endocardial ablation(s) and are not at exceedingly high risk of complications associated with epicardial access as part of the combined approach.

### Ventricular arrhythmia recurrence

Our analysis revealed that the combined endo-epicardial approach was associated with a significant reduction in risk of VT recurrence as compared to the endocardial-only approach (pooled OR of 0.47 and HR of 0.39) based on Bayesian and frequentist analyses respectively. Overall, these results demonstrate a substantial and durable reduction in the recurrence of VT in patients with ARVC after a combined endo-epicardial approach. Furthermore, the NNT of 6 with the combined approach also seems to be an acceptable trade-off for efficacy.

### Major complications

Historically, an epicardial ablation approach has been utilized as a second-line option in patients with previously unsuccessful endocardial ablation as part of a stepwise approach. This approach is considered reasonable, given the increased risk associated with proceeding with epicardial ablation as a first line strategy [27, 28]. The findings of our meta-analysis, which observed pooled OR of 6.28 (with Bayesian analysis) and 3.19 (with frequentist analysis) are statistically significant and indicators of higher risk of complications with the combined approach. We acknowledge that there were variations in the adjudication of procedural complications across the studies. While some studies had defined them as procedural complications alone, especially those resulting in permanent injury, or requiring intervention or hospitalization, whereas other studies had assessed the incidence of complications over a relatively longer follow-up period. The latter included the thromboembolic risk, vascular and pericardial complications. Despite this variability in adjudication of complications, sensitivity analysis supported that no single study significantly affected the overall results of our analyses. Our findings were also in alignment with the general findings of major complications associated with an epicardial approach, which are estimated to be in the range of 6–8% [29, 30]. Among these are major bleeding events such as: hemopericardium, damage to coronary arterial system and phrenic nerve injury. These sets of complications are within the expected spectrum of complications given the proximity of coronary arterial system and course of phrenic nerve encountered on the epicardial aspect during mapping and ablation.

### Clinical implications

Previously published literature has suggested that the adverse histopathological changes that form the underlying ventricular substrate in patients with ARVC, typically progress from the epicardial aspect to the endocardium [31, 32]. In a study by Basso et al., wherein the investigators had analyzed data from the time of autopsy of patients with ARVC and observed that histopathological changes included myocardial atrophy, cellular apoptosis and eventual fibrofatty replacement. It was also noted that these spectra of changes were localized to the subepicardium [29]. As expected, from a clinical perspective, a greater burden of adverse histopathological changes is observed to be associated with a greater burden of VTs during catheter ablation.

It is also worth discussing that both the mapping and ablation of the abnormal substrate in patients with ARVC can have distinct challenges, such as the unclear influence of fibrofatty infiltration on the voltage cut-offs during electroanatomical mapping [4]. Regional variations in the thickness of the RV and perivalvular areas in patients with ARVC can also pose difficulties which impede effective, transmural delivery of RF from the endocardial aspect. Additionally, despite the thin endocardium, often the critical isthmi of VTs tend to be localized to the in the mid-myocardial region. In combination, we speculate that these impediments are likely more pronounced in an endocardial-only approach which is typically adopted as a first-line option for ARVC patients with drug-refractory VT [31]. Although endocardial ablation is generally superior to anti-arrhythmia drugs for ARVC, the overall success rates of endocardial VT ablation for ARVC remain relatively low. One earlier study reported a success rate of 50% for long-term freedom from VT with an endocardial-only approach [13]. Similarly, an overall low success rate of 50% was again reported in a relatively recent study by Chaumont et al. However, the investigators in this study had also observed that the patients older than 45 years and those who were non- inducible at the end of index ablation had a greater success [26].

In line with the histopathological findings, the mapping studies have also observed that the epicardial aspect has more extensive abnormalities (fractionated and delayed electrograms, VT circuits) and greater distribution of overall low-bipolar voltage areas in ARVC patients. Based on procedural complexity and patient anatomy, it is also possible that certain areas cannot be accessed by an endocardial approach alone and therefore a combined approach might lend better success [5]. Furthermore, there is increasing evidence that in patients with non-ischemic cardiomyopathies, VT circuit often tends to involve the epicardium [33]. A combined approach also might lend superior success in targeting the mid-myocardial substrate from the endocardial aspect and that using an epicardial approach by itself might not obviate the need for endocardial ablation to achieve non inducibility of certain mid-myocardial circuits of VT [34]. Therefore, it is reasonable to believe that the combined approach is more effective in addressing the extensive arrhythmogenic substrate that might extend from epicardium towards endocardium.

Taken together, these results highlight the risks and benefits of an endocardial ablation first compared to a primary endocardial-epicardial approach. It is also worth mentioning that pericardial complications (such as bleeding, and risk of pericardial adhesions) with an upfront epicardial approach might make it challenging to obtain subsequent epicardial access if needed [35]. It is reasonable to consider the importance of careful patient selection for a stepwise, endo-epicardial approach which weighs both the decreased rates of VA recurrence against the increased risk of complications. In terms of decision-making, a collective approach based on imaging and mapping findings as well as balancing risk of complications can help guide whether to proceed with the combined approach upfront or in a stepwise manner.

An important consideration when interpreting our findings is the heterogeneity in procedural strategies across the included studies. There was considerable variation in the timing of epicardial access, ranging from upfront combined approaches to different staged strategies, as well as in mapping techniques. Differences in the use of substrate-based versus activation-guided ablation, along with variability in operator experience and institutional practice may have influenced both the rates of recurrence and complications. In particular, more extensive substrate modification with upfront combined approaches may contribute to improved arrhythmia control, whereas staged strategies are often applied in more complex or refractory cases, potentially introducing bias in outcome comparisons. Similarly, differences in mapping strategy and ablation endpoints may affect lesion completeness and long-term efficacy. Variability in operator expertise and VT ablation volume at a given center might also play a role in determining procedural safety. Taken together, these factors likely contribute to the observed variability in both efficacy and major complication rates. Therefore, the pooled estimates in our analyses should be interpreted in the context of these differences.

Earlier meta-analyses have shown that a combined endo-epicardial approach can reduce VA recurrence in ARVC, largely based on study-level data analyzed using conventional frequentist methods [36, 37]. In the present analysis, we sought to further build on this existing literature by using a Bayesian framework that allowed inclusion of both single-arm and comparative studies, thereby increasing the overall sample size. Additionally, reconstruction of individual patient data from Kaplan–Meier curves allowed us to examine recurrence free survival over time, thus offering a more clinically meaningful perspective on treatment durability.

## Limitations

We acknowledge several limitations of our analysis. There was at least a moderate degree of heterogeneity in terms of the definition of procedural complications in some of the studies included in our analysis. In order to enrich the currently available evidence, we included single-arm studies along with double-arm studies. Due to the lack of a comparison group in single arm studies, this could have introduced at least some degree of bias in our overall analysis. The survival analysis based on reconstructed individual patient data (IPD) from Kaplan–Meier curves represents an approximation rather than true patient-level data and may be influenced by the quality and resolution of the digitized curves. Only the studies which had reported Kaplan–Meier curves could be included, resulting in a reduced sample size and despite broadly comparable follow-up durations, some variability still persisted. Hence, these findings should be interpreted with caution. Additionally, there was variability in the timing and indication for epicardial access across studies, including upfront combined approaches and different staged strategies (failure-driven, recurrence-driven, selective and hybrid). These differences in procedural approach may have contributed to variation in both ventricular arrhythmia recurrence and complication outcomes. Differences in operator experience and center-specific practices might have also influenced the outcomes. Moreover, the relatively low number of complication events across studies limits the precision of effect estimates and contributes to variability between analytical approaches for the outcome of major complications. Hence, these findings should be interpreted with caution. Lastly, the included studies were observational in design, comprising both prospective and retrospective cohort and are therefore subject to inherent risks of confounding and selection bias.

## Supplementary Information

Below is the link to the electronic supplementary material.

Supplementary Material 1 (DOCX 2.71 MB)

## Data Availability

All data used in this network meta-analysis were obtained from previously published studies, as cited in the manuscript. No new data were generated, and no unpublished data were used.

## Abbreviations

ARVC: Arrhythmogenic right ventricular cardiomyopathy
VA: Ventricular arrhythmia
VT: Ventricular tachycardia
MC: Major complications
IPD: Individual patient data
CI: Confidence interval
CrI: Credible interval
HR: Hazard ratio
MAE: Mean absolute error
RMSE: Root mean square error
MaxAE: Maximum absolute error

## Declarations

AM: Consulting: Biotronik, Mariana AI, Vektor Medical Inc. EKH: Consulting: Biotronik, Boston Scientific, Future Cardia
